# Infective Arthritis: Bacterial 23S rRNA Gene Sequencing as a Supplementary Diagnostic Method

**DOI:** 10.2174/1874285800802010085

**Published:** 2008-06-13

**Authors:** Claus Moser, Keld Andresen, Anne Kjerulf, Suheil Salamon, Michael Kemp, Jens Jørgen Christensen

**Affiliations:** 1 Department of Bacteriology, Mycology, and Parasitology, Statens Serum Institut, Copenhagen, and the Departments of Clinical Microbiology; 2 Copenhagen University Hospital, Rigshos; 3 Copenhagen University Hospital, Herlev; 4 Vejle Sygehus, Denmark

**Keywords:** Synovial fluid, 23S rRNA, PCR, bacteria, infective arthritis

## Abstract

Consecutively collected synovial fluids were examined for presence of bacterial DNA (a 700-bp fragment of the bacterial 23S rRNA gene) followed by DNA sequencing of amplicons, and by conventional bacteriological methods. One or more microorganisms were identified in 22 of the 227 synovial fluids (9,7%) originating from 17 patients. Sixteen of the patients had clinical signs of arthritis. For 11 patients molecular and conventional bacterial examinations were in agreement. *Staphylococcus aureus*, *Streptococcus dysgalactiae subspecies equisimilis* and *Streptococcus pneumoniae*, were detected in synovial fluids from 6, 2 and 2 patients, respectively. In 3 patients only 23S rRNA analysis was positive; 2 synovial fluids contained *S. dysgalactiae subspecies equisimilis* and 1 *S. pneumoniae*). The present study indicates a significant contribution by PCR with subsequent DNA sequencing of the 23S rRNA gene analysis in recognizing and identification of microorganisms from synovial fluids.

## INTRODUCTION

Establishment of molecular methods for detection of microbiological etiologies of infectious diseases, including sequencing of the genes coding for bacterial rRNA, has provided new tools for identification of the etiology of infections [1, 2]. The molecular methods are of special interest, when an etiology with fastidious bacteria difficult to culture or slow growing bacteria may be suspected [2, 3]. Moreover, culture independent diagnostics are to prefer if antibiotic treatment has been initiated before sampling of material for microbiological testing [4].

Infective arthritis is a severe and painful condition which can be complicated by tissue destruction and permanent damage of the joint, and in addition, the mortality rate for in-hospital infective arthritis ranges from 7% to 15%, despite antibiotic use [5]. Furthermore, a substantial proportion of synovial fluids are culture-negative even from patients with typical signs of infective arthritis, suggesting a role of fastidious or slow growing pathogens in such clinical presentations [6]. Since antibiotic treatment is possible in the case of a bacteriological etiology, rapid and correct diagnosis of the pathogen is mandatory [7, 8].

In the present study, 227 non-selected synovial fluids, both from native and artificial joints, consecutively sent to the laboratories of clinical microbiology in three different hospitals in the Copenhagen area of Denmark, were analysed for presence of a 700-bp segment of the bacterial 23S rRNA gene in parallel to conventional analysis by microscopy and culture.

## MATERIALS AND METHODS

*Specimen sampling and patients:* All consecutively unselected synovial fluids, irrespective of tentative diagnosis, sent to the Departments/Unit of Clinical Microbiology, at Copenhagen University Hospitals, Rigshospitalet and Herlev Hospital, and at Statens Serum Institut, were included in the study. A substantial number of the synovial fluids were expected to be sterile. If possible the synovial fluids were divided before analysis. Otherwise, PCR was performed on the synovial liquid remaining after conventional microbiological examinations. The remaining fluid was kept at -20(C until molecular analysis.

Patient data, on the 17 patients from whom synovial fluids contained bacterial DNA and/or gave growth of bacteria, were obtained from the patient records. Six of the patients were females and 11 were males. The median age was 64-years with a range from 1 to 80 years (Table **1**). Sixteen of the patients had clinical signs of arthritis, while status was unknown for one. Seven patients had an arthroplasty and 3 patients suffered from rheumatoid arthritis, of which 2 patients had both. In 5 of 7 patients arthroplasties had to be removed and in 1 patient with arthroplasty lifelong antibiotic treatment was initiated.

* Conventional microbiological examinations:* Synovial fluids were centrifuged at 1,590 x *g* for 10 min. The supernatants were discarded, and the pellet resuspended in the remaining liquid. The suspensions were plated on a 5% horse-blood agar (SSI, Copenhagen, Denmark) and a chocolate agar with heat-treated defibrinated horseblood (SSI) and cultured for 2 days at 37 (C in a 5% CO_2_-enriched atmosphere. In addition, material was plated on agar selective for Gram negatives (SSI) and cultured for two days at 37 (C. Material on an additional chocolate plate was cultured in an anaerobic atmosphere and observed for bacterial growth at day 2 and 5. Each sample was analysed by microscopy after staining with Gram stain and methylene blue staining at a 1000x magnification.

*DNA extraction:* One ml of each sample was centrifuged at 16.000 x *g* for 10 min and the pellet resuspended in 200 µl of sterile PBS. The DNA was extracted using the QIAamp DNA Blood Mini Kit according to the manufacturers recommendations (Qiagen).

*PCR assay:* The primers used for the amplification of 23S rDNA were Uni-F (5`-TAA CGG TCC TAA GGT AGC GAA ATT-3`) and Uni-R (5`-GAT AGG GAC CGA ACT GTC TCA CG-3`), which produced a 700-bp fragment of 23S rDNA. The PCR mixture (50 µl total volume), contained 1 x PCR buffer, 2.5 mM MgCl_2_, 200 µM each deoxynucleoside triphosphate, 200 µM each primer and 1.25 U of Taq DNA polymerase (Qiagen). One and 5 µl samples were tested in PCR. The amplification profile was 95 ^o^C for 15 min, followed by 40 cycles at 94 ^o^C for 30 s, 60 ^o^C for 30 s and 72 ^o^C for 30 s. Amplicons were resolved on a 2% agarose gel, visualized by ethidium bromide under UV illumination and digitally recorded.

*DNA sequencing:* Both DNA strands of the amplicons were sequenced on an ABI PRISM 3100 Avant Genetic Analyzer (applied Biosystems) using Uni-F and Uni-R as sequencing primers and the BigDye v. 3.1 kit (Applied Biosystems). Sequencing data were edited using the SeqScape Software (Applied Biosystems) and only data from overlapping sequences were used in the data processing. Using default parameters in the BLAST search engine, the edited sequencing data were then compared to sequences deposited in the “Bacteria” NCBI database (National Center for Biotechnology Information,www.ncbi.nlm.nih.gov/BLAST.Blast files were stored electronically and later evaluated with respect to %/number of identities, MaxScore (bits) and E-values for the best and the next best matches [6, 9].

## RESULTS

*Microbiological examinations:*For 11 patients molecular and conventional bacterial examinations were in agreement (cases 1,2,4,7-12,15,17) (Table **1**). *Staphylococcus aureus*, *Streptococcus dysgalactiae subspecies equisimilis* and *Streptococcus pneumoniae*, were detected in synovial fluids from 6, 2 and 2 patients, respectively. Coagulase negative staphylococci (CNS) were grown in addition to *S. dysgalactiae subspecies equisimilis* from one patient (case 7) and their presence interpreted as a contamination. In one patient as well *Citrobacter freundii* as *Pseudomonas aeruginosa* was grown and 23S rRNA analysis demonstrated *C. freundii*, but polymicrobial infection could not be excluded. In 3 patients each only culture (cases 13,14,16) or 23S rRNA analysis (cases 3,5,6), respectively, was positive. Of the solely culture positive cases, *S. aureus* was grown in 2 synovial fluids and CNS in 1 synovial fluid, while the 23S rRNA analysis examinations, in the cases with *S. aureus* grown, were insufficient because of less than 1 ml fluid examined/inhibition of PCR reaction. Of the solely 23S rRNA analysis positive synovial fluids 2 contained *S. dysgalactiae subspecies e-quisimilis* and 1 *S. pneumoniae*. For the 23S rRNA positive synovial fluids, differences in percentage of similarity and Maxscore between best and second best taxon match resulted in good favor of the identified microorganism.

In synovial fluids from nine patients microorganism were detected by microscopy, either streptococci (n = 4; cases 1-4) or staphylococci (n = 5; cases 8-12). As well culture as 23S rRNA gene analysis were positive in these instances, except in one patient (case 3) only having pneumococci detected by the 23S rRNA gene analysis. No microorganisms were seen in synovial fluids from 8 patients. From three patients* S. dysgalactiae subspecies equisimilis*, was detected by 23S rRNA gene analysis, but only grown from 1 patient and from 3 patients *S. aureus* was grown, but only detected by 23S rRNA gene analysis in synovial fluid from 1 patient. The last 2 patients (case 16 and 17) had CNS grown and a suspected polymicrobial etiology by both methods, respectively.

## DISCUSSION

The aim of the present study was to investigate to what extent PCR of the bacterial 23S rRNA gene and DNA sequencing of the amplicon could add to microbiological diagnosis obtained by culturing of synovial fluid. PCR and DNA sequencing resulted in identification of an infecting organism in three patients, whom were negative by culture. This is equivalent to an 17% increase in positive rate. Though the number of examined synovial fluids preferably could be higher, PCR and DNA sequencing contributed importantly in the microbiological diagnosis of patients suspected of infective arthritis in agreement with recent literature [10-12]. Both, among synovial fluids with and without a positive microscopy for microorganisms 23S rRNA gene analysis added to defining a bacterial etiology. However, culture is still mandatory as the primary analysis giving the possibility of susceptibility testing [10]. Especially in children positive rates have been increased when using improved culture methods, i.e. inoculation of synovial fluid into blood-culture bottles, and when using universal eubacterial ribosomal DNA (rDNA) PCR methods [10, 11]. Ferroni [10] even recommends if the culture is negative to carry out a universal PCR or a PCR targeted to the main bacterial etiologies responsible for infective arthritis.

In a study on diagnosis of joint infection by PCR on swabs or synovial fluids from 154 patients, no significant gain was achieved as compared to conventional culturing [13]. In particular, no exotic bacteria were identified. However, in another study on material dislodged from retrieved prostheses, bacterial DNA was identified by PCR in 72% of 120 patients as compared to positive culture in 22%. This indicates that the incidence of prosthetic joint infections is grossly underestimated [14]. However, the bacterial DNA was not identified further.

The main etiologic agents of infective arthritis are Gram-positive cocci such as *Staphylococcus aureus*, beta-hemolytic streptococci and to a lesser extend *Streptococcus pneumoniae* [11], which also was the case in this study. Positive findings by PCR were considered of clinical significance in the present study, since only significant pathogens were identified, and clinical signs of infection were present in all 15 cases. In children *Kingella kingae* has been recognized as the etiologic agent in approximately 15% of cases in which a microorganism is recognized [11, 12]. Only few children were included in our study, which may explain that no arthritis cases caused by *K. kingae* were found. Detection of microorganisms which are difficult to culture is considered a major advantage for the new molecular diagnostic methods [1, 2]. Streptococci are to a certain degree troublesome in culturing, especially if antibiotics have been administered prior to sampling. Indeed, in the 3 23S rRNA gene analysis positive, but culture negative samples, *S. dysgalactiae subspecies equisimilis *(n=2) and *S. pneumoniae* (n=1) were detected supporting superior detection in such situations by PCR for 23S the rRNA gene analysis [15].

Molecular diagnostic methods allows detection of microorganisms that are difficult to culture, including bacteria considered as exotic. In other sites of infection, e.g. infective endocarditis *Coxiella burnetii*, *Bartonella henselae* and *Tropheryma whipplei* has been detected and identified by DNA amplification and sequencing [16]. In agreement with previous reports, the present study did not find exotic bacteria in joint fluids. This, probably reflects that such organisms are indeed very rare causes of infective arthritis [7, 8].

Advantage of examining for bacterial ribosomal genes is predicted in the cases where antibiotics have been given prior to isolation of the samples. Such benefit of using the molecular method was achieved for 2 of the PCR positive, but culture negative samples. In 1 case (case 3) streptococci were identified by microscopy. This patient was admitted with pneumonia and sepsis and pneumococci were cultured from the blood, and the patient eventually died. In another case (case 6) a new sampling of synovial fluid three weeks later was culture positive with an identical microorganism. This patient had an arthroplasty related infection, and was treated with penicillin and dicloxacillin. Finally, the arthroplasty was removed and the patients had an arthrodesis. In the third patient (case 5) synovial fluid was positive for bacterial DNA of the 23S rRNA gene,but culture negative; additional findings were a positive blood-culture with *S. dysgalactiae* and sign of ostitis with several illuminations on a bone scintigraphy.

There is good agreement in identification of microorganisms when using phenotypic and ribosomal gene sequencing methods [17]. Also, in the present study no conflicting results were seen in identifications done on phenotypic characterization or 23S rRNA gene analysis. The same organisms were identified when both methods were positive, except the 2 cases where more than 1 microorganism was identified in the same synovial fluid. Two synovial fluids both harboured two microorganisms identified by culture, whereas PCR for 23S rRNA genes only identified 1 microrganism in each of the 2 samples. Detection of 2 or more significant pathogens requires separation of DNA products after the PCR reaction, forenstance by denaturating gradient gel electrophoresis. In the case where as well *C. freundii *as* P. aeruginosa* were isolated both pathogens were thought to be of significance, whereas the coagulase-negative staphylococci detected in case 10 were considered as a contaminant.

In conclusion, the present study indicates a significant contribution by use of bacterial 23S rRNA gene analysis in detection and identification of microorganisms from synovial fluids. Continued suspicion of infective arthritis despite of negative cultures should lead to the use of molecular diagnostics

## Figures and Tables

**Table 1.** Data on 17 Patients Suspected of Infective Arthritis with Bacteria Detected/Identified in Synovial Fluids by 23S rRNA gene Analysis and/or Conventional Microbiological Detection (Microscopy/Culture)

Case	Sex, Age in Years	Affected Joint	23S rRNA-analysis	Conventional Culture	Microscopy
1	M, 73	Right elbow	*S. pneumoniae*	*S. pneumoniae*	Streptococci seen
2	F, 1	Elbow	*S. pneumoniae*	*S.pneumoniae*	Streptococci seen
3	M, 55	Right hip	*S. pneumoniae*	No growth	Streptococci seen
4	M, 78	Left knee	*S dysgalactiae subsp. equisimilis*	*S. dysgalactiae*	Streptococci seen
5	M, 65	Knee	*S. dysgalactiae subsp. equisimilis*	No growth	Microorganisms not seen
6	M, 66	Left knee	*S. dysgalactiae subsp. equisimilis*	*No growth*	Microorganisms not seen
7	M, 57	Left elbow	*S. dysgalactiae subsp. equisimilis*	*S. dysgalactiae* and CNS	Microorganisms not seen
8	F, 66	Right elbow	*S. aureus *	*S. aureus *	Staphylococci seen
9	F, 80	Left shoulder	*S. aureus*	*S. aureus*	Staphylococci seen
10	F, 75	Left knee	*S. aureus*	*S. aureus*	Staphylococci seen
11	F, 78	Knee	*S. aureus*	*S. aureus*	Staphylococci seen
12	M, 64	Right knee	*S. aureus*	*S. aureus*	Staphylococci seen
13	M, 20	Right ankle	*IE*	*S. aureus*	Microorganisms not seen.
14	M, 50	Elbow	*IE*	*S. aureus*	Microorganisms not seen
15	M, 55	Left knee and ankle, right wrist	*S. aureus*	*S. aureus*	Microorganisms not seen.
16	M, 43	Left knee	IE	CNS	Microorganisms not seen.
17	F, 44	Left knee	*C. freundii^a^*	*C. freundii P. aeruginosa*	Microorganisms not seen.

S. pneumoniae: Streptococcus pneumoniae; S. dysgalactiae subspecies equisimilis: Streptococcus dysgalactiae subspecies equisimilis; S. aureus Staphylococcus aureus; C. freundii: Citrobacter freundii; P. aeruginosa: Pseudomonas aeruginosa CNS: Koagulase negative staphylococci.; IE: Incomplete examination (less than 1 ml synovial fluid and/or inhibition of PCR reaction) a: polymicrobial etiology could not be excluded.
